# Expression of Human Mutant Huntingtin Protein in *Drosophila* Hemocytes Impairs Immune Responses

**DOI:** 10.3389/fimmu.2019.02405

**Published:** 2019-10-16

**Authors:** Yu-Hsien Lin, Houda Ouns Maaroufi, Emad Ibrahim, Lucie Kucerova, Michal Zurovec

**Affiliations:** ^1^Biology Centre of the Czech Academy of Sciences, Institute of Entomology, Ceske Budejovice, Czechia; ^2^Faculty of Science, University of South Bohemia, Ceske Budejovice, Czechia

**Keywords:** Huntington's disease, immunity, infection, *Drosophila* melanogaster, phagocytosis, cytokines, antimicrobial peptide (AMPs)

## Abstract

The pathogenic effect of mutant HTT (mHTT) which causes Huntington disease (HD) are not restricted to nervous system. Such phenotypes include aberrant immune responses observed in the HD models. However, it is still unclear how this immune dysregulation influences the innate immune response against pathogenic infection. In the present study, we used transgenic *Drosophila melanogaster* expressing mutant HTT protein (mHTT) with hemocyte-specific drivers and examined the immune responses and hemocyte function. We found that mHTT expression in the hemocytes did not affect fly viability, but the numbers of circulating hemocytes were significantly decreased. Consequently, we observed that the expression of mHTT in the hemocytes compromised the immune responses including clot formation and encapsulation which lead to the increased susceptibility to entomopathogenic nematode and parasitoid wasp infections. In addition, mHTT expression in *Drosophila* macrophage-like S2 cells *in vitro* reduced ATP levels, phagocytic activity and the induction of antimicrobial peptides. Further effects observed in mHTT-expressing cells included the altered production of cytokines and activation of JAK/STAT signaling. The present study shows that the expression of mHTT in *Drosophila* hemocytes causes deficient cellular and humoral immune responses against invading pathogens. Our findings provide the insight into the pathogenic effects of mHTT in the immune cells.

## Introduction

Huntington's disease (HD) is an inherited neurodegenerative disorder caused by an abnormal expansion of CAG trinucleotide in the Huntingtin (*htt*) gene. Mutant HTT protein (mHTT) contains an extended polyglutamine tract encoded by 40 to over 150 CAG repeats, which causes cytotoxicity and leads to neurodegeneration; this results in involuntary movement, cognitive impairment, and psychiatric abnormalities (1). Although many clinical symptoms of HD are related to neuronal dysfunction, emerging evidence indicates that the expression of mHTT in non-neuronal cells of the brain or in the peripheral tissues also contributes to the pathogenesis of HD (2). Abnormal phenotypic effects caused by the dysfunction of non-neuronal cells have been described in cardiac cells, muscles, the endocrine system, adipose tissue, testes and immune cells of HD patients, and also in mouse HD models (2, 3).

Abnormalities related to the immune system were observed in a number of studies of HD patients (4). The expression of mHTT in both brain and peripheral immune cells (microglial and myeloid cells) induces the NF-κB signaling pathway which elevates levels of pro-inflammatory cytokines and chemokines, leading to systemic inflammation (5). In addition, macrophages isolated from HD model mice exhibited migration deficits, and microglia showed a delayed response to laser-induced injury in the brain (6). Although several studies proposed that the immune cell response is impaired in HD, this phenomenon is still poorly characterized in relation to host responses to pathogens. One recent study reported increased proliferation of a parasite, *Toxoplasma gondii*, in HD model mice, causing premature mortality and thus suggesting that expression of mHTT in immune cells may suppress immune responses (7).

*Drosophila melanogaster* has been long-term established as a HD model. *In vivo* experiments have revealed that the ectopic overexpression of mutant human *htt* (exon 1 with expanded CAG repeats) in the neural tissue of transgenic flies causes neurodegeneration (8, 9). The mechanisms of cellular pathology observed in the HD flies seem similar to those in human patients, including the suppression of mitochondrial function, transcriptional dysregulation, and neuronal apoptosis (10, 11). Genetic screening for disease modifiers in HD model flies led to the identification of the effects of sumoylation and HSP70 chaperone machinery on neurodegeneration. The subsequent confirmation that these pathways are involved in the pathology of human patients validates the *Drosophila* model for investigating HD (12, 13). Furthermore, since the tissue-specific expression of transgenes in *Drosophila* can be easily controlled using the UAS-Gal4 system, *Drosophila* have also been used to study the effects of HD on non-neuronal cells, including glial cells, photoreceptors, cardiac cells, and salivary glands (14–18).

The present study aimed to survey the physiological impact of mHTT expression in *Drosophila* hemocytes. We used the *Drosophila* UAS-Gal4 system to express mHTT with hemocyte-specific drivers and investigated the effect of mHTT on survival, hemocyte development, and susceptibility to pathogens. We also expressed mHTT in a *Drosophila* macrophage-like cell line, S2 cells, and assessed the effect of mHTT on phagocytic activity, ATP levels, antimicrobial peptides, and production of cytokines. Our results suggest that the expression of mHTT in hemocytes does not directly affect survival but causes immune dysregulation, which leads to an impaired immune response against pathogenic invasion.

## Results

### Expression of mHTT in Hemocytes Did Not Affect Larval Viability but Decreased the Number of Circulating Hemocytes

**Figure 1:**
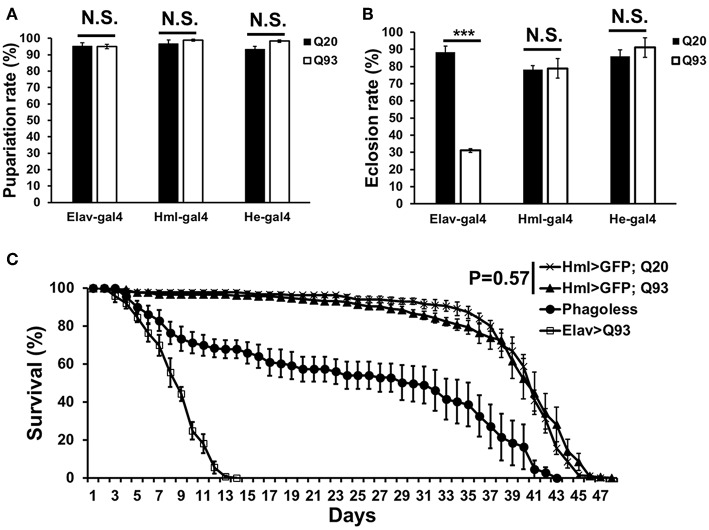
The viability assays of mHTT-expressing flies under the control of the pan-neuronal driver (*elav*-*gal4*) and hemocyte drivers (*hml-gal4* and *he-gal4*). **(A)** The effect of mHTT expression on pupariation (survival to pupal stage). **(B)** Eclosion (survival to adulthood) and **(C)** adult longevities were measured in control Q20 and mHTT Q93. All the experiments performed in at least six independent replicates. Data are presented as averages ± SEM. *P*-values for pupariation and eclosion rate using Student's *t-*test, ^***^P < 0.001, N.S., not significant. Significance analysis for longevity curve using weighted log-rank test.

In order to characterize the effects of mHTT in *Drosophila* hemocytes, we used a tissue-specific UAS-Gal4 system by expressing wild-type human HTT (Q20) or mutant HTT (Q93) under the control of a pan-neuronal driver, *elav-gal4*, or hemocyte drivers, *hml-gal4*, and *he-gal4*. The flies devoid of plasmatocytes (*phago*^*less*^) generated by expressing pro-apoptosis genes, *rpr* and *hid* with *hml-gal4* were used as negative control (19, 20). The results showed that the ectopic expression of Q93 with the pan-neuronal driver (*elav-gal4*) decreased both the eclosion rate and the longevity of the adult flies, but not the rate of pupariation (Figure 1). The expression of Q20 and Q93 with both hemocyte drivers (*hml-gal4* and *he-gal4*) had no effect on pupariation and eclosion rates. Furthermore, the differences in longevity between Q20 and Q93 flies were not significant, and their survival rate was higher than *phago*^*less*^ flies (Figure 1C). These results indicated that the hemocyte-specific expression of mHTT did not influence fly viability, unlike its expression in the brain.

**Figure 2:**
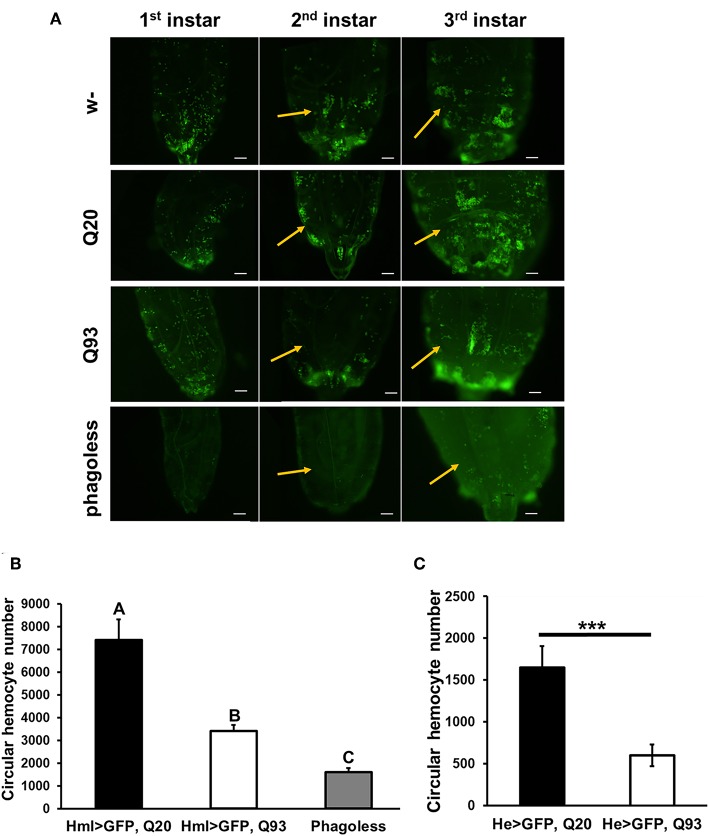
Ectopic expression of mHTT decreased hemocyte numbers. **(A)** Microscope images indicated the decreased number of circulating and sessile hemocytes in mHTT-expressing second-instar larvae. Quantification of hemocytes by ectopic co-expression of HTT with GFP using *hml-gal4* **(B)** or *he-gal4* **(C)**. *Phago*^*less*^ flies with hemocyte ablation (*hml* > *UAS-rpr, hid*) were used as a negative control. The number of the circular hemocyte corresponded to the total number of GFP positive cells in 25 μL of collected sample. At least five independent replicates were analyzed. Data are presented as averages ± SEM. Significances were analyzed by ANOVA with Fisher LSD *post-hoc* test **(B)**, and the significant differences among treatment groups are marked with different letters (*P* < 0.05). Student's *t*-test was used for **(C)**, ^***^P < 0.001.

Although the expression of mHTT did not affect fly survival, we observed a significant decrease in the number of circulating hemocytes. In first-instar larvae, the number of hemocytes differed significantly only in the *phago*^*less*^ flies (Figure 2A). However, a reduced amount of circulating and sessile hemocytes was apparent in the Q93 mutants from second-instar larvae. As shown in Figure 2B, the circular hemocyte numbers in Q93 larvae were still higher than in *phago*^*less*^ flies, showing about 50% of the numbers observed in the Q20 control (Figure 2B). These results showed that the expression of mHTT with two different hemocyte-specific drivers reduced the number of hemocytes.

### Expression of mHTT in Hemocytes Impaired the Immune Response to Parasites

**Figure 3:**
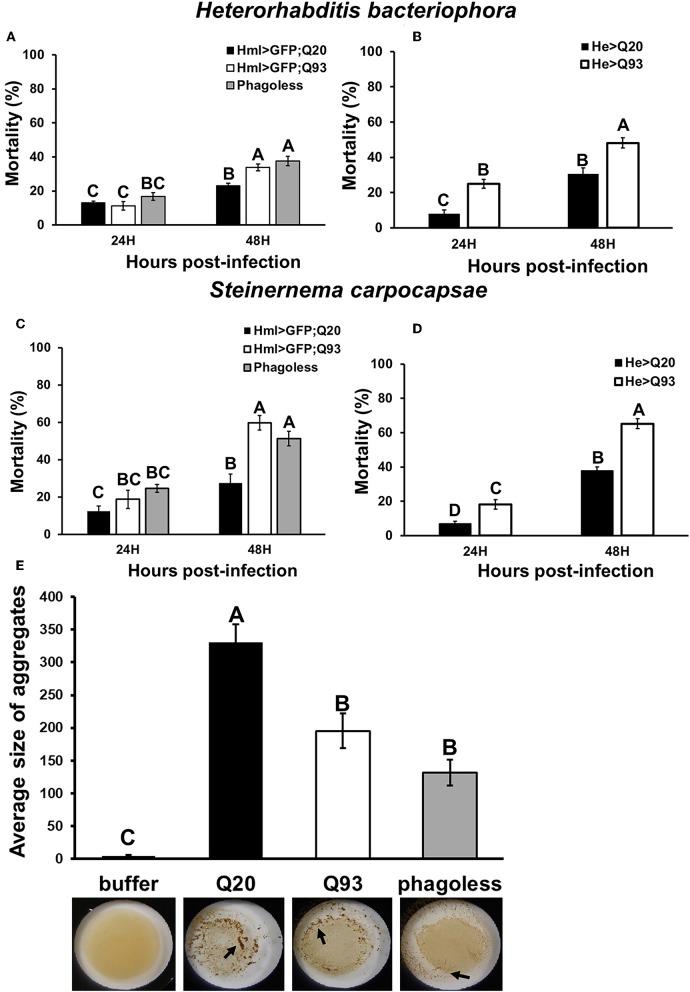
Immune challenge with entomopathogenic nematode infection and clotting assay. Larvae expressing mHTT Q93 or HTT Q20 with *hml-gal4* or *he-gal4* hemocyte drivers were infected with *H. bacteriophora* **(A,B)**, or *S. carpocapsae* **(C,D)**. Mortality was calculated 24 and 48 h after infection. *Phago*^*less*^ flies with hemocyte ablation (*hml* > *UAS-rpr, hid*) were used as negative control. Bead aggregation assay was used for assessing the clotting activity **(E)**. Hemolymph was collected from Q93, Q20, and *Phago*^*less*^ (*hml-gal4*) larvae, mixed with a bead suspension, and the aggregates were quantified by ImageJ software. All the experiments were performed in five to six independent replicates. Data are presented as averages ± SEM. Significances were analyzed by ANOVA with Fisher LSD *post-ho*c test; significant differences among treatment groups are marked with different letters (*P* < 0.05).

To examine whether mHTT expression in *Drosophila* hemocytes affects the innate immune response and whether such larvae are still able to restrain parasite development, we tested the sensitivity of such flies to entomopathogenic nematode and parasitoid wasp infections, which are two *Drosophila* pathogenic models for examining the cellular immune response (21, 22). Early third-instar larvae expressing mHTT (Q93), wild-type HTT (Q20) or *phago*^*less*^ were infected with nematode species, *Heterorhabditis bacteriophora* or *Steinernema carpocapsae*, which contain the bacterial symbionts *Photorhabdus luminescens* and *Xenorhabdus nematophila*, respectively. Mortality was calculated at 24 and 48 h post-infection. As shown in Figure 3, both *phago*^*less*^ and Q93 larvae displayed significantly higher mortality than Q20 controls. Previous studies revealed that the formation of hemolymph clot is an important innate immune response against entomopathogenic nematode infection in *Drosophila* (23, 24). To determine whether the expression of mHTT in the hemocytes caused clotting defects, we used an established bead aggregation assay (24, 25). Compared to the larvae expressing normal HTT (Q20), the hemolymph collected from mHTT (Q93)-expressing larvae displayed poor bead aggregation similar to *phago*^*less*^ larvae (Figure 3E). This results indicated that the expression of mHTT suppresses the clotting activity and thus increases the susceptibility to nematode infection.

**Figure 4:**
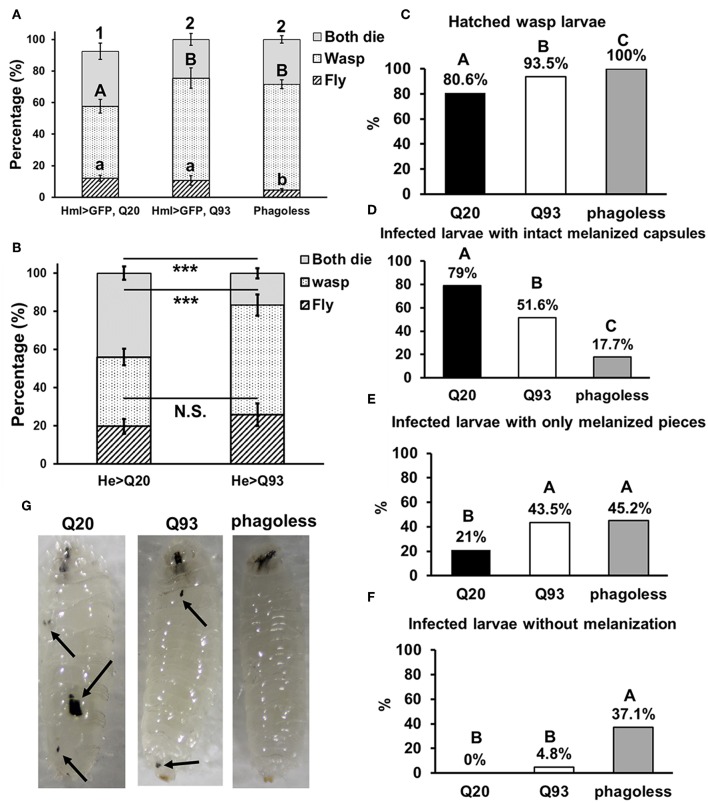
Immune challenge with parasitoid wasp infection and encapsulation activity assay. Larvae expressing mHTT Q93 and HTT Q20 with *hml-gal4* **(A)** or *he-gal4* **(B)** hemocyte driver were infected with parasitoid wasp, *L*. *boulardi*. *Phago*^*less*^ flies (which underwent hemocyte ablation) were used as negative control. Thirty infected larvae were collected and the numbers of eclosed flies and wasps were calculated, indicating the number of flies overcoming the wasp infection and the number of wasps successfully escaping the fly's immune reaction, respectively. Data are presented as average ± SEM with more than 10 biological replicates. The significances of results for **(A)** were analyzed by ANOVA with a Fisher LSD *post-hoc* test; different letters on the treatment group indicate significant differences at *P* < 0.05. The significances of results for **(B)** were examined using Student's *t*-test, ^***^P < 0.001, N.S., not significant. For assessing the encapsulation activity, the numbers of fly larvae containing larvae of parasitic wasps **(C)**, intact melanized capsules **(D)**, melanized pieces **(E)** as well the numbers of the infected larvae without melanization **(F)** were recorded. The significances of the results were analyzed by Mann-Whitney *U*-test (paired); significant differences among treatment groups are marked with different letters (*P* < 0.05). The photos show examples of intact melanized capsule in Q20 larvae, melanized pieces in Q20 and Q93 larvae and infected *phago*^*less*^ without melanization reaction **(G)**.

Similarly, we infected *Drosophila* larvae with a parasitoid wasp, *Leptopilina boulardi* and calculated the number of emerged fly and wasp adults. The number of eclosed *Drosophila* adults was not significantly different between Q20 and Q93 driven by *hml-gal4* and *he-gal4*, while *phago*^*less*^ showed lower eclosion rates than both Q20 and Q93 (Figures 4A,B). However, the number of emerged wasps were significantly higher in both Q93 and *phago*^*less*^ flies, thus indicating that a greater number of wasps overwhelmed the immune reaction of Q93 hosts and successfully developed to adult stage. In addition, the higher number of wasp eggs successfully hatched in both Q93 and phagoless larvae (Figure 4C); these results indicated that Q93 and *phago*^*less*^ larvae have less efficient immune reaction against wasp infection. Since the encapsulation and melanization are major defense mechanisms against parasitoid wasp infection, we quantified the number of the melanized capsules to assess the immune activity after 72 h post-infection. We found that there were more intact melanized capsules in Q20 larvae (79%) than in those expressing Q93 (51.6%) or in *phago*^*less*^ (17.7%) (Figures 4D,G left). We also observed a higher amount of melanization pieces in Q93 or *phago*^*less*^ individuals than in Q20 larvae (Figures 4E,G middle). The formation of such defective capsules was described previously in immune-deficient mutant flies (26). Moreover, 37% of the infected *phago*^*less*^ larvae formed no melanization capsules compared to Q20 (0%) or Q93 (4.8%) infected larvae (Figures 4F,G right). These results could explain a lower proportion of *phago*^*less*^ adults successfully eclosed after wasp infection (Figures 4A,B). Taken together, our results suggest, that mHTT expression impairs the innate immune reactions to nematode and parasitoid wasp infections due to the deficient cellular immune responses such as clot formation and encapsulation.

### Reduced Phagocytic Activity and ATP Levels in mHTT Cells

To find out whether mHTT expression could cause a detrimental effect on hemocyte functions, we expressed mHTT or wild-type HTT in *Drosophila* S2 cells. The S2 cell line consists of macrophage-like cells with phagocytic activity and the ability to produce antimicrobial peptides (AMPs) (27). We transfected the cells with four different recombinant constructs encoding green fluorescent protein (GFP) fused to HTT repeats under an inducible metallothionein promoter. We created stable cell lineages and confirmed that the S2 cells expressed HTT-fusion proteins by observing the GFP. As shown in Supplemental Figure 1, most of the cells in all cell lineages were positive for the fluorophore. Furthermore, the cells containing the mHTT Q46, Q72, and Q97 constructs (all except wild-type Q25) showed formation of mHTT aggregates.

**Figure 5:**
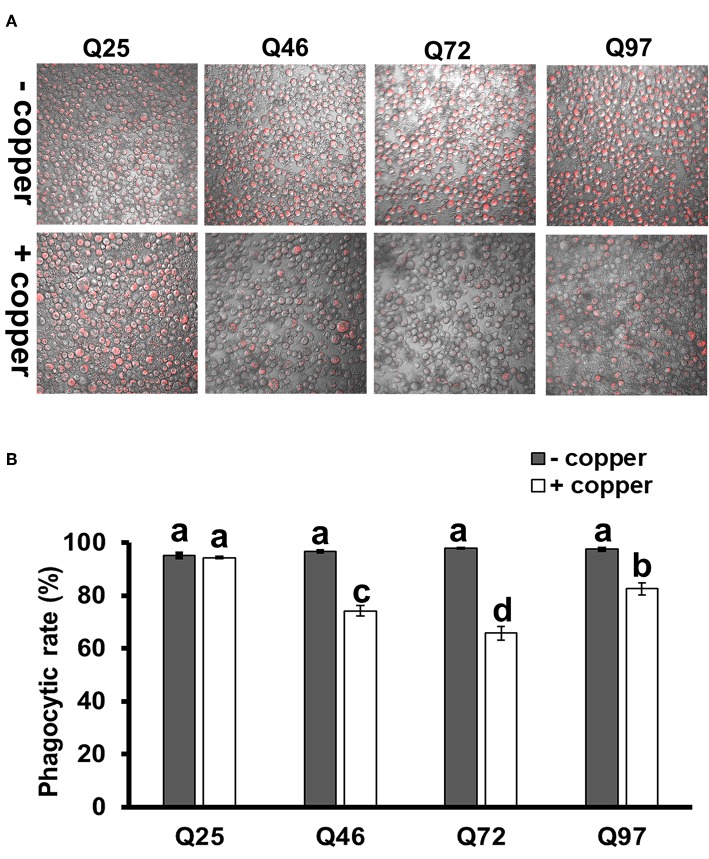
Impairment of phagocytic activities in mHTT-expressing S2 cells. S2 cell lineages expressing wild-type HTT (Q25) and mHTT (Q46, Q72, and Q97) were treated with pHrodo Red *E*. *coli* for 8 h. **(A)** Fluorescence microscope images show the decreased intensity of red fluorescence signals in mHTT-expressing cells. **(B)** The phagocytic rate was calculated as the percentage of cells showing a red fluorescence signal to the total number of cells in each image. Each treatment was performed in three independent replicates with (+copper) or without (–copper) CuSO_4_ induction. Data are presented as average ± SEM. Significances were analyzed by ANOVA with Tukey HSD *post-hoc* test; different letters on the treatment group indicate significant differences at *P* < 0.05.

We further treated the HTT-expressing cells with *E*. *coli* particles conjugated by pH-sensitive dye (pHrodo) to examine their phagocytic activity. This causes bright fluorescence to be visible after particle engulfment in the acidic environment of phagolysosome. The results showed that after inducing mHTT expression, the fluorescence signals were significantly lower in Q46, Q72, and Q97 mHTT-expressing cells but not in cells expressing wild-type Q25 HTT (Figure 5A). Quantification of the cells containing fluorescent signals showed a significant reduction (20–30%) of fluorescent-positive cells in mHTT-expressing cells compared to the control cells (Q25) (Figure 5B), thus supporting the hypothesis that expression of mHTT in immune cells impairs phagocytic activity.

**Figure 6:**
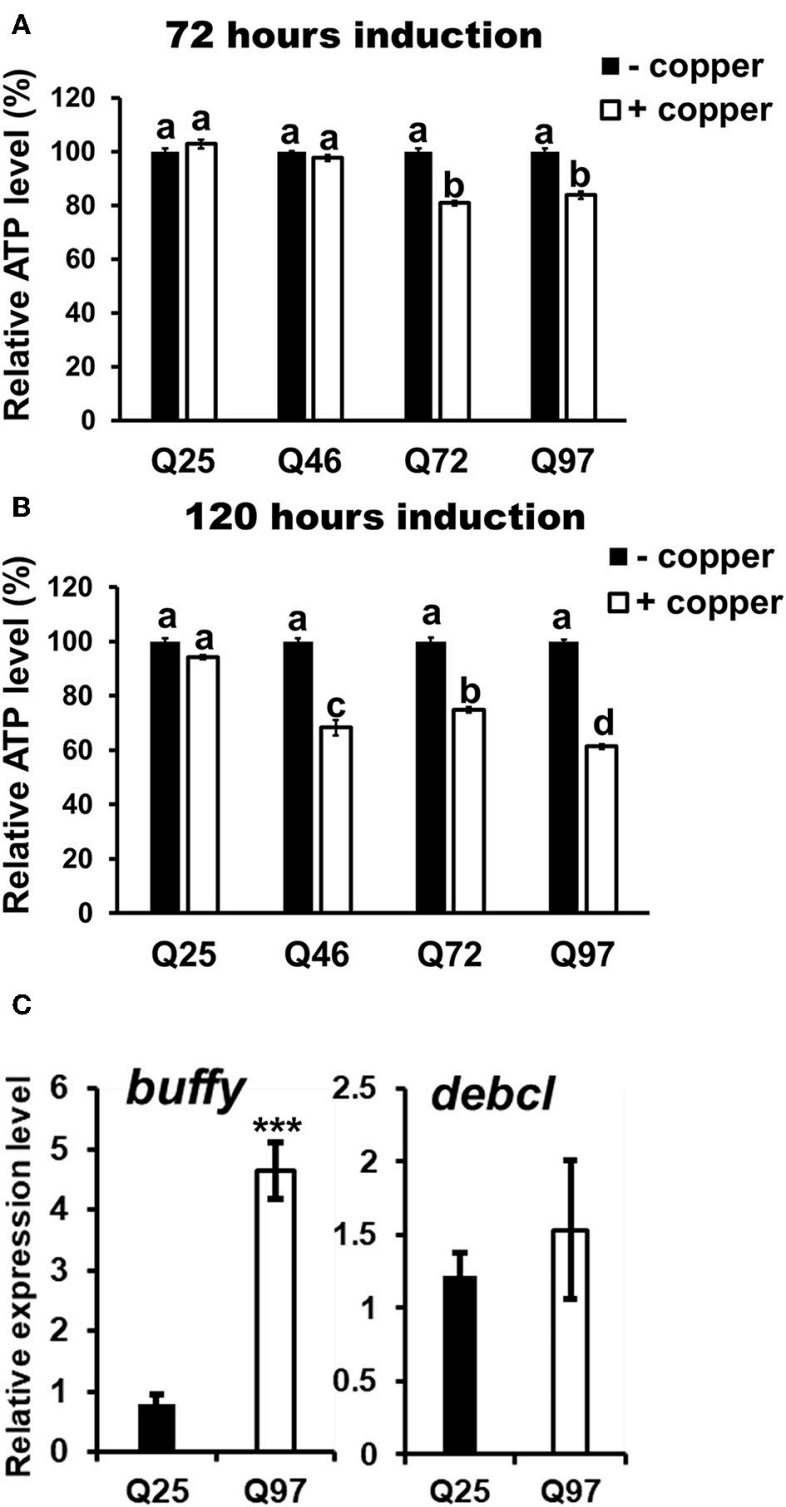
Decrease of ATP level and alternation of Bcl-2 protein expression in mHTT-expressing S2 cells. ATP levels of HTT- and mHTT-expressing S2 cells (5 × 10^4^ cells) were measured after 72 **(A)** and 120 h **(B)** of copper induction. Each treatment was performed in five independent replicates with (+copper) or without (–copper) copper induction. Data are presented as average ± SEM. Significances were analyzed by ANOVA with Tukey HSD *post-hoc* test; different letters on the treatment group indicate significant differences at *P* < 0.05. The mRNA levels of *Drosophila* Bcl-2 proteins, *buffy* and *debcl* were measured in Q25 HTT and Q97 mHTT-expressing S2 cells after 120 h of induction **(C)**. All the expressions were normalized to *rp49* transcript and Q25 control (ΔΔCT). Data are presented as average ± SEM from three independent replicates. *P*-values were determined using Student's *t*-test, ^***^P < 0.001.

The phagocytic capacity of immune cells has been associated with mitochondrial activity (28–30); mHTT has been shown to cause impairment of energy metabolism and mitochondrial dysfunction in human peripheral blood cells (31). To test whether mHTT can also impair the energy metabolism of *Drosophila* immune cells, we measured the ATP levels in S2 lineages after mHTT induction. The results showed that ATP levels significantly decreased in cells expressing Q72 and Q97 mHTT after 72 h of induction (Figure 6A). The ATP levels in cells expressing Q46, Q72, and Q97 mHTT were further reduced after 120 h of induction (Figure 6B). This indicated that the expression of mHTT reduces ATP levels, which may further limit the cellular immune responses against pathogenic infection.

The studies in human and mouse have demonstrated that the expression of Bcl-2 family proteins associated with mitochondrial dysfunction is activated by mHTT expression (32). To assess whether the level of *Drosophila* Bcl-2 proteins is also altered by mHTT expression, we compared the transcription levels of two of *Bcl-2* genes, *buffy* and *debcl*, in Q25- and Q97- expressing S2 cells (Figure 6C). We found that *buffy* expression is five times higher in Q97- than in Q25- expressing cells, but we did not detect any significant difference in *debcl* mRNA level. Different from pro-apoptotic function of *debcl, buffy* was suggested to play an anti-apoptotic role under stress conditions which is similar to mammalian Bcl-2 proteins (33, 34). We conclude that the alternation of ATP synthesis and *buffy* expression indicate the abnormality of mitochondrial function in mHTT expressing cells, and the induction of *buffy* might be a protective mechanism for preventing the cell death caused by mitochondrial dysfunction.

### Upregulation of Cytokines Expression and Downstream JAK/STAT Signaling in mHTT Expression Cells

**Figure 7:**
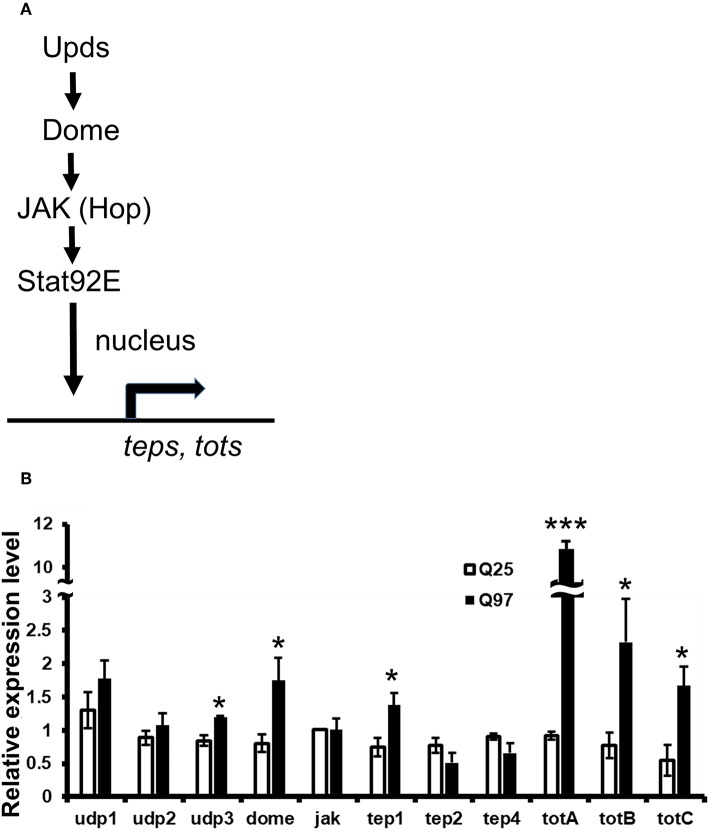
Activation of cytokine expression and JAK/STAT signaling in the mHTT-expressing S2 cells. **(A)** Schematic representation of the interaction between Upds and JAK/STAT pathway. **(B)** The gene expression of cytokines (*udp1-3*), *dome, jak*(*hop*), and JAK/STAT downstream target genes (*teps* and *tots*) were measured in Q25 HTT and Q97 mHTT-expressing S2 cells after 120 h of copper induction. All the expressions were normalized to *rp49* expression and Q25 control (ΔΔCT). Data are presented as average ± SEM from three independent replicates. *P*-values using Student's *t*-test, ^*^P < 0.05, ^***^P < 0.001.

It has been reported that the level of cytokines and chemokines are abnormally increased in the plasma of HD patients (35). Consistently, the production of cytokines from monocytes and macrophages of HD patients have shown hyper-activation after lipopolysaccharide stimulation (36). To test whether mHTT has a similar effect in *Drosophila*, we used Schneider 2 (S2) cells and measured the effect of mHTT expression on three *Drosophila* cytokines, *upd1, upd2*, and *upd3*, as well as *dome, jak* (*hop*) and downstream targets of JAK-STAT signaling (Figure 7A). The results showed that the expression of cytokine *upd3* is significantly increased in Q97 mHTT-expressing cells compared to Q25 controls (Figure 7B). In addition, the expression of *dome* receptor and four downstream targets, *tep1, totA, totB*, and *totC* were also significantly increased in Q97-expressing cells. These results indicated that the expression of mHTT induced the production of cytokines and activates JAK/STAT signaling.

### Decreased Antimicrobial Peptide (AMP) Production in Response to Bacteria

**Figure 8:**
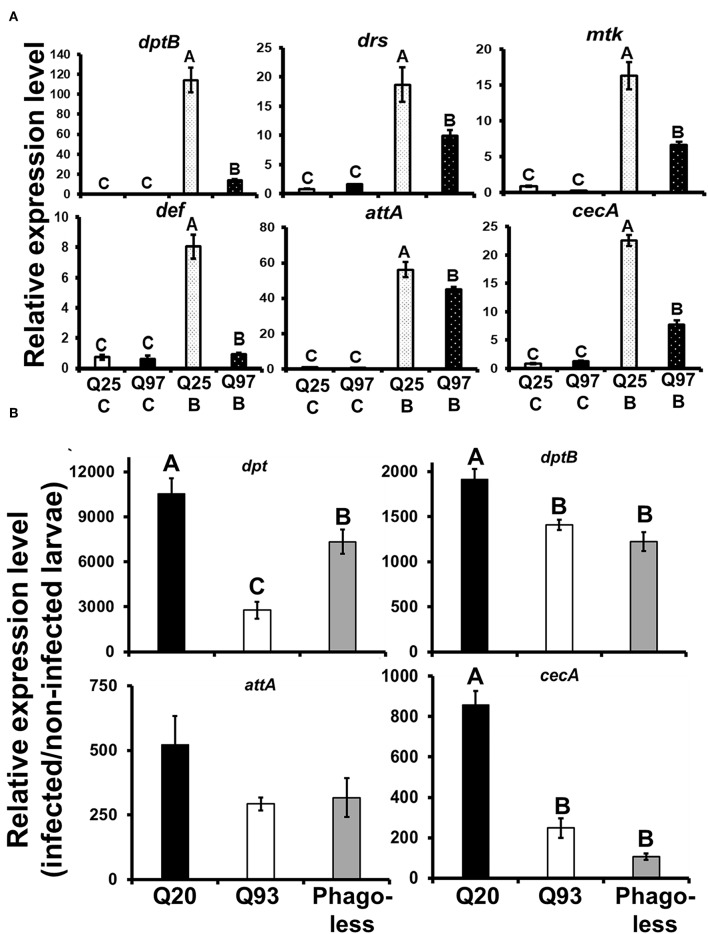
Suppression of antimicrobial peptides (AMPs) induction after bacterial treatment in the mHTT-expressing S2 cells and larvae. **(A)** Q25 and Q97-expressing S2 cells were incubated with *E. coli* and *M. luteus* (Q25_B and Q97_B) or without bacteria (Q25_C and Q97_C) for 8 h, and the expression levels of AMPs was measured. The AMPs expressions were normalized to *rp49* expression and Q25 control. **(B)** Larvae expressing mHTT Q93 or HTT Q20 under *hml-gal4* hemocyte drivers as well as *Phago*^*less*^ mutants were infected with ECC15-GFP and their expression of AMPs was determined after 8 h. The expressions were normalized to *rp49* transcripts and non-infected controls. The AMPs expression levels of non-infected controls for each genotype were set to one. All the data are presented as average ± SEM from three independent replicates. The significances were analyzed by ANOVA with Fisher LSD *post-hoc* test; different letters on the treatment group indicate significant differences at *P* < 0.05.

*Drosophila* Toll and Imd pathways control the humoral immune response against invasive microorganisms by regulating the induction of downstream AMP genes in both hemocytes and the fat body (37). To examine whether AMP induction was affected by mHTT expression in *Drosophila* S2 cells, we treated mHTT-expressing cells with a mixture of heat-inactivated Gram-negative bacteria, *Escherichia coli*, and Gram-positive bacteria, *Micrococcus luteus*. The induction of AMPs was assessed using qPCR. As shown in Figure 8, there was no difference in the expression of AMPs between Q25 and Q97 in the absence of bacterial treatment. In contrast, all AMP genes were significantly induced in both Q25- and Q97-expressing cells at 8 h after bacterial treatment. However, AMP induction levels were significantly lower in cells expressing Q97 (Figure 8A). We further assessed the AMP expression levels under *in vivo* condition after infecting larvae with phytopathogenic bacteria, *Erwinia carotovora carotovora 15* (Ecc15). We examined the expression levels of *dpt, dptB, attA*, and *cecA* which were known as being highly induced after Ecc15 infection (38). Our results of larval infections showed that except for *attA*, the induction levels of *dpt, dptB*, and *cecA* in Q93 or *phago*^*less*^ larvae were significantly lower than in Q20 controls (Figure 8B). These results confirm that the induction of AMPs in response to bacteria was significantly suppressed in mHTT-expressing cells or larvae.

## Discussion

Peripheral immune dysregulation is considered as one of the clinical features of HD pathogenesis (39). Previous studies in mice and HD patients have suggested that mHTT expression in immune cells accelerates the neurodegenerative process. The activation of pro-inflammatory products in mHTT-expressing microglial cells elevate the reactive oxygen species (ROS) and cause neuroinflammation, which contributes to neurodegeneration (5, 40). Genetic ablation or pharmacologically-blocked cannabinoid receptor 2 (interleukin-6 regulator), as well as drug suppression of the cytokine-responsive kynurenine pathway, can both slow neurodegeneration and improve the phenotype of R6/2 HD mice (41, 42). Since the expression of mHTT in HD mice and human patients is ubiquitous, it is still unclear whether mHTT expression in blood cells directly contributes to the lethal effect of HD. The present study examined mHTT expressed specifically in *Drosophila* blood cells and assessed its impacts on development and longevity (Figure 1). We found that the expression of mHTT in hemocytes did not cause mortality or a shortening in life span, which is in contrast to expression in the brain. Our results, therefore, suggest that expression of mHTT in immune cells does not directly contribute to mortality.

A reduced proliferation of immune cells has been observed in *T. gondii*-infected HD mice, in which the expansion of CD8^+^ T-cells in the spleen and brain was significantly suppressed during infection (7). Our results showed that the expression of mHTT in flies with hemocyte-specific drivers causes a significant reduction in the number of circulating hemocytes (Figure 2), and this decrease might be caused by dysfunction of mitochondria (Figure 7). The mitochondrial abnormalities resulting in metabolic dysregulation in peripheral blood cells of HD patients increase oxidative damage and suppress their anti-oxidant capacity (40). The activation of caspase-3 and caspase-9 in lymphoblasts of HD patients increases apoptosis under stress conditions (43).

The mHTT-expressing larvae revealed a higher susceptibility to wasp and nematode infections and this phenotype was caused by defects of clot formation and encapsulation (Figures 3, 4). It has been shown that wasp egg recognition by circulating plasmatocytes and their differentiation to lamellocytes for further encapsulation are important processes of the immune response against wasp invasion in *Drosophila* (44). The production of clotting components from hemocytes also contributes to wound healing and melanization, which are important against nematode or wasp infections (24, 45). mHTT-expressing macrophages and monocytes from HD mice and patients also showed migration defects toward an inflammatory stimulus (6). Hemocyte migration and adhesion are important factors for the development of embryonic macrophages, as well as successful wound healing and encapsulation during wasp infection (46). Furthermore, decreased phagocytic activity toward bacterial particles (Figure 5) and a suppressed induction of antimicrobial peptides (Figure 8) can also contribute to immune deficiency against the bacterial symbionts of nematodes (23, 47).

A previous study showed that macrophages isolated from HD patients and R6/2 mice displayed increased phagocytosis when incubated with fluorescent polystyrene beads (48). Our results seemingly differ because we observed reduced phagocytic activity of the *Drosophila* macrophage-like cells expressing different mHTT fragments (Figure 5). Unlike their approach, we tested phagocytic activity using *E*. *coli* particles with a pH-sensitive fluorescent dye that can accurately confirm phagosome formation and initiation of the phagolysosome acidification. However, similar to their results, we found that S2 cells expressing mHTT were able to initiate phagocytosis. We tested this by treating the S2 cells with heat-inactivated *E*. *coli* labeled with DNA-specific fluorescent dye (without pH-sensor); the results showed that mHTT-expressing cells were indeed able to engulf *E*. *coli* (Supplemental Figure 2). Thus, our results suggest that mHTT-expressing cells were unable to complete the process of phagocytosis to final phagolysosome acidification. In addition, a defective actin function has been reported in HD mouse immune cells leading to failure of membrane ruffling (6), which supports our results since actin assembly is required to trigger engulfment and phagolysosome maturation for successful phagocytosis (49).

Consistent with previous observations in HD mice and patients, we also found that *Drosophila* hemocyte cytokine *udp3* was upregulated in mHTT-expressing cells (Figure 8). *Udp3* binds to the JAK/STAT signaling receptor, Dome, and initiates phosphorylation cascades which translocate the transcription factor, Stat92E, into the nucleus and activates downstream target genes (Figure 7A) (50). Two selected downstream target genes, *tep1* and *totA*, were highly expressed in mHTT-expressing cells (Figure 7B). Notably, we found that the induction of antimicrobial peptides was significantly suppressed in mHTT-expressing cells after bacterial treatments, which has not yet been observed in other HD models. It is known that several human antimicrobial peptides are expressed in blood cells including neutrophils and macrophages (51). Since the transcriptomic analysis in HD blood cells has shown dysregulation of transcription in large genomic regions (52), further studies will be needed to understand whether the production of antimicrobial peptides is impaired in the blood cells of patients or HD mice during infection.

**Figure 9:**
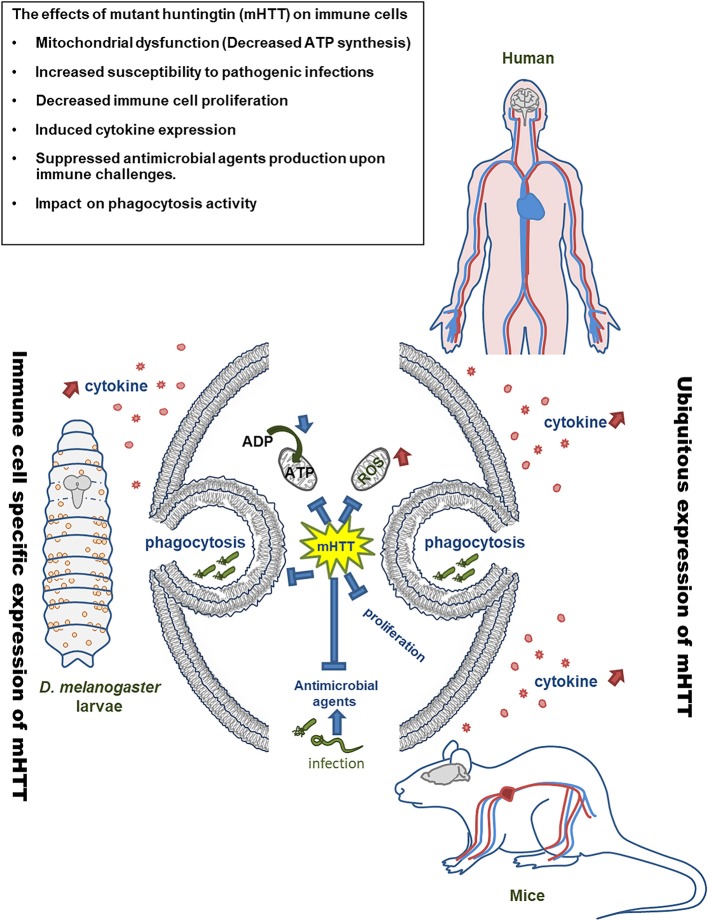
Model of mHTT effects on *Drosophila* and mammalian immune cells. The expression of mHTT in hemocytes of *Drosophila* displays similar effects on the immune cells of HD patients and mice.

In summary, the present study demonstrates immune dysregulation in flies expressing mHTT in hemocytes (Figure 9). This expression does not directly cause a lethal effect, although it does reduce the number of circulating hemocytes and decrease ATP levels. Cytokine expression and downstream JAK/STAT signaling are activated upon mHTT expression, which has also been observed in HD patients and mice. In addition, the induction of antimicrobial peptides as well as the immune response against different pathogenic infections are impaired in mHTT-expressing *Drosophila* cells. The present study introduces a system for studying the tissue-specific effects of mHTT in *Drosophila* immune cells. Further studies can be applied to clarify the molecular interaction between mHTT and antimicrobial peptide pathways (Toll and IMD signaling) as well as the mechanisms of phagocytosis suppression.

## Materials and Methods

### Fly Stocks

Flies were reared at 25°C on standard cornmeal medium. The fly strains used were UAS-Q20Httexon1^111F1L^ and UAS-Q93Httexon1^4F132^ obtained from Prof. Lawrence Marsh (UC Irvine, USA) (8), which contain 20 (wild-type) and 93 (mutant HTT) polyglutamine repeats, respectively. The pan-neuronal driver, *elav-gal4*[C155], and hemocyte drivers, *he-gal4* and *hml-gal4*, were obtained from Bloomington *Drosophila* Stock Center and Dr. Tomas Dolezal (University of South Bohemia), respectively (53–55). Hemocyte-ablated flies (*phago*^*less*^) were used as negative controls and were generated by overexpressing pro-apoptotic proteins (*UAS-rpr, -hid*) with *hml-gal4* (19, 20, 56).

### Developmental and Longevity Assay

Thirty first-instar larvae collected from a juice plate were transferred into vials to measure the number of pupae and adults for each replicate. For the longevity assay, about 20–30 newly emerged male adults were collected for each replicate and maintained at 29°C. Q93 expression driven by pan-neuronal driver, *elav-gal4* and *phgo*^*less*^ flies were used as positive controls for longevity assay. Since expression of Q93 driven by *elav-gal4* (X chromosome insertion) results in high mortality of male progeny (dosage compensation) (57), female progeny were used for recording the longevity. The number of dead flies was counted every day. All the experiments were performed in at least six independent replicates.

### Circulating Hemocyte Counting

Circulating hemocytes were obtained from larvae by cuticle tearing in Ringer's buffer with thiourea to prevent melanization (25 μl of buffer per 6 larvae). The number of hemocytes expressing GFP (*hml-gal4* or *he-gal4* > *UAS-gfp*) were counted using a hemocytometer. At least five independent replicates were analyzed for each genotype.

### Parasitoid Wasp Infection, Eclosion, and Encapsulation Assay

*Leptopilina boulardi* parasitoid wasps were obtained from Dr. Jan Hrček (Biology Center CAS) and maintained by infecting wild-type *Drosophila* larvae. For the wasp infection assay, forty larvae (second instar) were transferred onto a dish containing cornmeal food, and three female wasps were then placed onto the dish and allowed to attack for 72 h. After infection, 30 infected larvae were collected from the dish and transferred into a vial containing cornmeal for each replicate. Each genotype was tested in at least 10 independent replicates. The total number of eclosed flies and wasps were calculated (26). For the encapsulation assay, the infected larvae were dissected 72 h post-infection and the number of larvae containing intact melanized capsules, broken melanized pieces as well as wasp larvae was recorded.

### Nematode Infection

Two nematode species, *Steinernema carpocapsae* and *Heterorhabditis bacteriophora*, were used in this study, under previously described maintenance conditions (58). For the infection assay, nematodes were combined with autoclaved water to achieve a concentration of 25 infective juveniles per 10 μl. Then, 10 μl of nematode suspension was applied to paper and placed in each wells of a 96-well plate. Individual larvae were transferred to each well where they stayed in contact with the nematodes, and the plate was covered with Parafilm. The infection was conducted at 25°C in the dark. Each experimental replicate consisted of 32 early third-instar larvae (72 h after egg hatching), and all experiments were done at least in five replicates. The number of dead larvae were counted after 24 and 48 h of infection (23, 24).

### Bead Aggregation Assay

The bead aggregation assay was described in our previous study (24). Briefly, 2.5 μl of hemolymph was collected from six late third-instar larvae, mixed with BSA-blocked bead suspension (tosylactivated Dynabeads M-280, Invitrogen), diluted in *Drosophila* Ringer (pH 6.0) in a well of cavity diagnostic slide (Thermo Scientific) and covered with round cover glass. Pictures were taken with a Nikon SMZ-745T stereomicroscope associated with a CANON EOS 550D. The images were analyzed and quantified with the ImageJ graphics software with the “Analyze Particles” module.

### Cell Culture

*Drosophila* Schneider 2 (S2) cells were grown at 25°C in Shields and Sang medium (Sigma) with 0.1% yeast extract, 0.25% peptone, and 10% heat inactivated fetal bovine serum. To generate stable lines expressing polyglutamine repeats, the S2 cells were transfected with four different Httex1-eGFP pMK33 plasmids (Q25, Q46, Q72, and Q97) containing copper-inducible metallothionein promoter (obtained from Dr. Sheng Zhang) (59).

### Phagocytosis Assay

After induction for 5 days (120 h) with 1 mM copper (CuSO_4_; Sigma), 100 μl of cell suspension (1 × 10^6^ cells/ml) was transferred to each well of a 96-well plate. Then, 100 μl of pHrodo Red *E. coli* (1 mg/ml; Thermo Fisher Scientific) was applied to each well for phagocytosis testing. After 8 h of treatment, the supernatant was removed, the cells were washed two times with 1× PBS, and 100 μl of fresh medium was applied. Cells were observed and photographed with a confocal microscope. From the images, the total number of cells and the number of cells displaying red fluorescence were counted. Three experimental repeats for each treatment were done for statistical analysis.

### ATP Measurement

Cells were treated with 1 mM copper for 3 days (72 h) and 5 days (120 h) to induce mHTT expression. Fifty microliter of a 1 × 10^6^ cells/ml solution (5 × 10^4^ cells) was transferred to each well of a 96-well plate. After removing the supernatant, 60 μl of CellTiter-Glo solution (Promega) was applied to each well for 10 min. Then, 50 μl of the mixture was transferred to each well of 96-well white plates and the intensity of luminescence was then measured. Five independent replicates for each treatment were performed for analysis.

### Bacterial Infection *in vitro* and *in vivo*

Five milliliter of S2 cells (1 × 10^6^ cells/ml) carrying copper-inducible Q25 HTT or Q97 mHTT transgenes were incubated in media containing 1 mM CuSO_4_ for 120 h in 60 mm tissue culture plates. After the induction, the cells were treated for 8 h with 1 ml of bacterial mixture containing *Escherichia coli* and *Micrococcus luteus* at an optical density (600 nm) of 1 (OD_600_ = 1) (37). The cells were then harvested for RNA extraction.

For the *in vivo* infection, late third instar larvae (96 h after egg hatching) were collected and transferred into a vial with 0.5 g instant *Drosophila* medium (Formula 4–24, Carolina Biological Supply) supplemented with 200 μl of bacterial suspension (OD_600_ = 50) *Erwinia carotovora carotovora 15*-GFP (ECC15-GFP) and 1,300 μl of distilled water. The larvae were collected for RNA extraction 8 h after the infection (19, 38).

### RNA Extraction

For *in vitro* experiments, S2 cells were washed with 1× PBS three times and harvested with 800 μl of RiboZol (VWR). Samples were preserved at −80°C until RNA purification. For *in vivo* experiments, 10 larvae were washed by distilled water and homogenized by the pestle motor (Kimble) in 200 μl of RiboZol (VWR Life Science). The sample were then preserved at −80°C for further RNA purification. RNA was isolated using NucleoSpin RNA columns (Macherey-Nagel) following the manufacturer's instructions and cDNA was synthesized from 2 μg of total RNA using a RevertAid H Minus First Strand cDNA Synthesis Kit (Thermo Fisher Scientific).

### qPCR and Primers

5× HOT FIREPol® EvaGreen® qPCR Mix Plus with ROX (Solis Biodyne) and an Eco Real-Time PCR System (Illumina®) were used for qPCR. The cDNA was diluted 50 times before use. Each reaction contained 4 μl of EvaGreen qPCR mix, 0.5 μl of forward and reverse primer (10 μM), 5 μl of diluted cDNA and ddH2O to adjust the total volume to 20 μl. The list of primers is shown in Supplemental Table 1. The expression level was calculated by using the (2^−ΔΔ*CT*^) method. The CT value of target genes were normalized to reference gene, ribosomal protein 49 (*rp49*).

### Statistical Analysis

Error bars show standard error of the mean throughout this paper. Significance was established using Student's *t*-test (N.S., not significant, ^*^P < 0.05, ^**^P < 0.01, ^***^P < 0.001) or one-way ANOVA analysis with Fisher LSD or Tukey HSD *post-hoc* test. The Mann–Whitney *U*-test was used for examing the significance of the data on wasp larval hatching and the host encapsulation activities (Figures 4C–F). For the statistical analysis of longevity curve, we used online tool OASIS 2 to perform the weighted log-rank test for determining significance (60).

## Data Availability Statement

The raw data supporting the conclusions of this manuscript will be made available by the authors, without undue reservation, to any qualified researcher.

## Author Contributions

Y-HL conceived the project, performed the experiments and prepared the manuscript. HM performed the hemocyte counting and imaging. EI performed the nematode infections. LK performed the clotting assay. MZ supervised the project and manuscript preparation.

### Conflict of Interest

The authors declare that the research was conducted in the absence of any commercial or financial relationships that could be construed as a potential conflict of interest.

## Supplementary Material

Supplemental Figure 1 Expression of four different lengths of HTT-GFP fusion proteins under a fluorescence microscope. mHTT-expressing cells (Q46, Q72, and Q97) showed significant mHTT aggregates after copper induction, while there was no aggregate formation in normal HTT-expressing cells (Q25).

Supplemental Figure 2 Phagocytosis assay in Q25- and Q97-expressing S2 cells with *E. coli* labeled by DNA-specific dye (Hoechst 33342). Cells expressing mHTT were able to initiate phagocytosis.

Supplemental Table 1 List of qPCR primers used in this study.
