# Supplementary figures and images for: Expression of Human Mutant Huntingtin Protein in Drosophila Hemocytes Impairs Immune Responses
Source: Front Immunol. 2019 Oct 16;10:2405. doi: 10.3389/fimmu.2019.02405 (PMC6805700; doi:10.3389/fimmu.2019.02405)

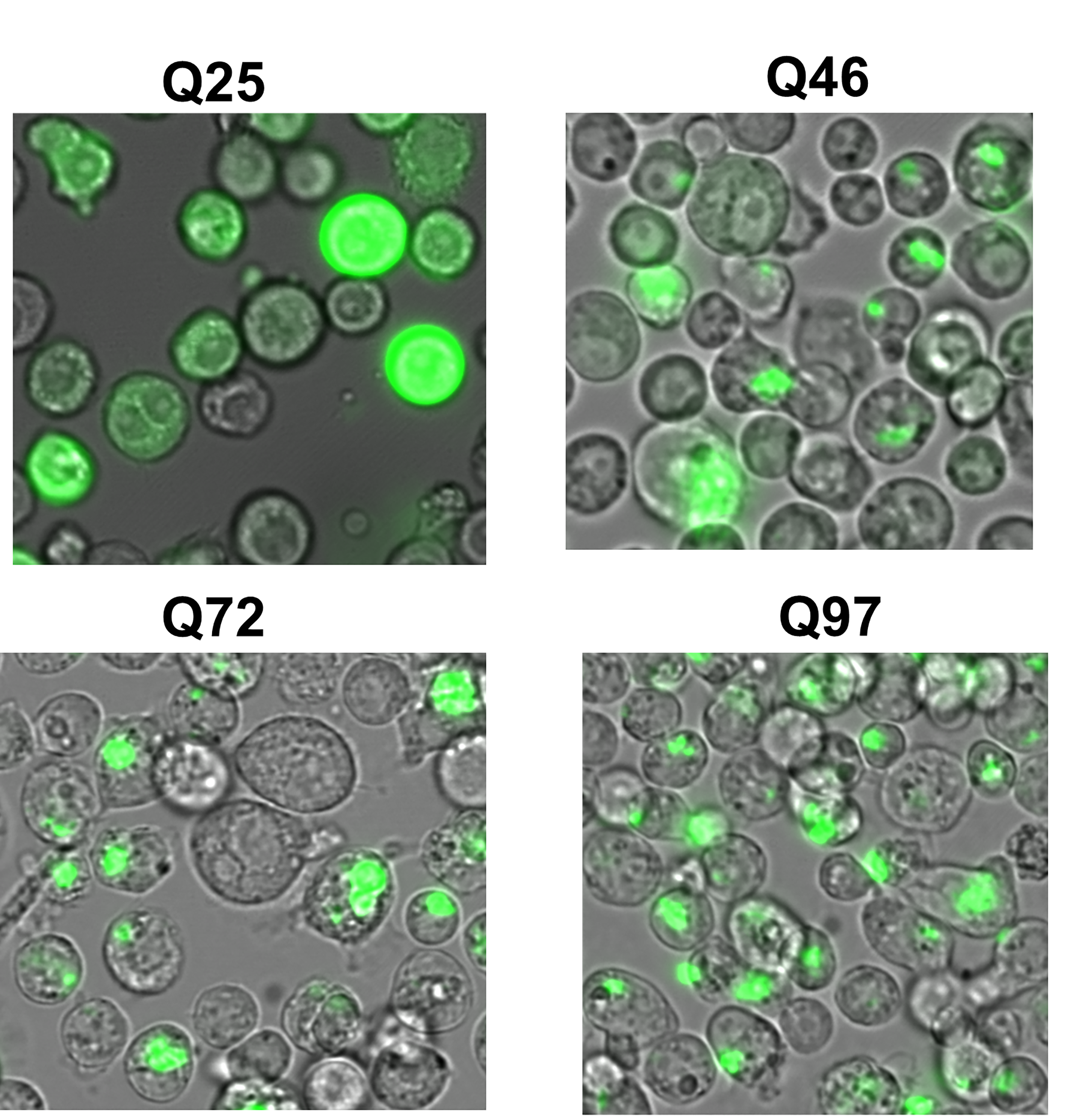

Supplement: Supplemental Figure 1 — Expression of four different lengths of HTT-GFP fusion proteins under a fluorescence microscope. mHTT-expressing cells (Q46, Q72, and Q97) showed significant mHTT aggregates after copper induction, while there was no aggregate formation in normal HTT-expressing cells (Q25). [file Image_1.TIF]

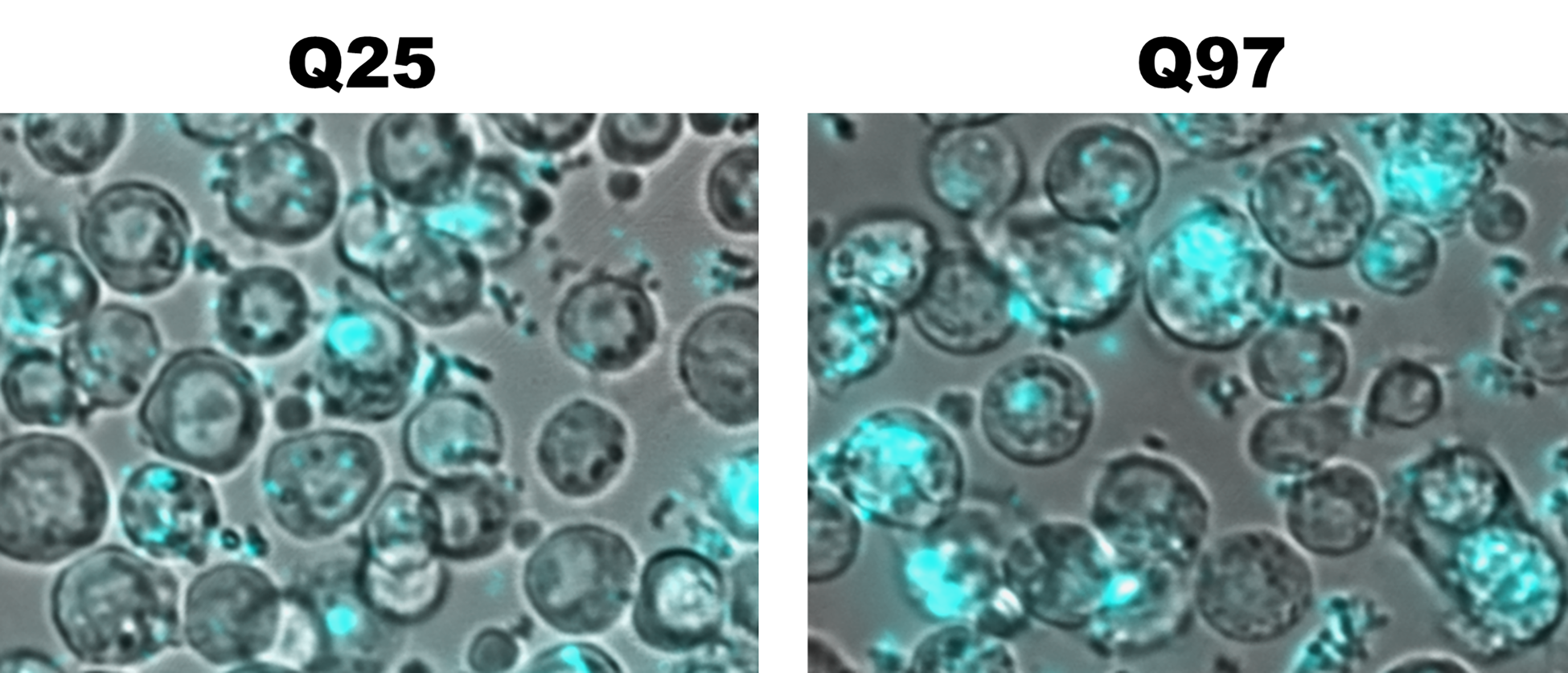

Supplement: Supplemental Figure 2 — Phagocytosis assay in Q25- and Q97-expressing S2 cells with E. coli labeled by DNA-specific dye (Hoechst 33342). Cells expressing mHTT were able to initiate phagocytosis. [file Image_2.TIF]
